# Correspondence

**Published:** 1869-01

**Authors:** H. C. Bartleson


Dear Register: In looking over a recent number of a Dental Journal, I noticed an article on "taking plaster impressions for partial cases," in which the author gave his method of procedure where the remaining teeth were long, with large crowns and small necks, or inclining toward each other.

Though his plan is very good, it occurred to me that the way I am in the habit of doing this thing may have the merit of being more simple and equally effectual. It is this: I select the cup most suitable and take an impression in wax in the usual manner, only that I make no attempt to get the roof of the mouth perfect, save where the posterior border of the plate is to come, and I draw it in the manner indicated to secure as perfect an impression of the teeth as possible. Then before the wax is cool, I trim it with a warmed knife, cutting off all that projected beyond the cup posteriorly, and shave away to the depth of an eighth of an inch all that which came in contact with the gum and hard palate, except at the posterior border of the impression, when I leave about a line's breadth, (to keep the plaster from running down the throat) and that which is close to the teeth, to preserve their form. The surface thus made I roughen by coarse scratches for the plaster to fasten to. Thus prepared, I re-introduce the now cold impression, to see that it has not been changed, and to observe the best manner it is accommodated to the teeth. I then pour the plaster where the wax has been removed to the amount that will slightly exceed the quantity of wax which was shaved away, and proceed in the usual manner of taking plaster impressions, pressing it firmly home. All excess of plaster will pass out over the gum where the teeth have been removed. I allow the plaster to become perfectly hard, having no fear of its being held by the teeth, as it does not come in contact with them. This method secures the accuracy of wax around the teeth, combined with the perfection of plaster for the palatine surface and gums.

Yours, truly, H. C. Bartleson.

